# Beyond Traditional Biometry: The Role of Fetal Kidney Length in Third Trimester Gestational Age Assessment

**DOI:** 10.7759/cureus.108887

**Published:** 2026-05-15

**Authors:** Dharmesh J Patel

**Affiliations:** 1 Obstetrics and Gynecology, Jawaharlal Nehru Medical College, Datta Meghe Institute of Higher Education and Research, Wardha, IND

**Keywords:** abdominal circumference (ac), biparietal diameter (bpd), femur length (fl), gestational age, head circumference (hc), kidney length

## Abstract

Aim and background: Fetal kidney length (FKL) demonstrates consistent and proportional growth throughout the third trimester, which makes it a promising candidate for accurate gestational age (GA) estimation. This study aims to elucidate whether FKL offers distinct advantages over existing methods or if it complements them effectively in enhancing the accuracy of GA assessment.

Methods: The present prospective cross-sectional study was conducted at the Department of Obstetrics and Gynecology at Acharya Vinoba Bhave Rural Hospital (AVBRH) from 2022 to 2024 among 100 normal pregnant patients attending Obstetrics and Gynecology services at AVBRH. Relevant clinical information, including maternal age, parity, medical history, and last menstrual period (LMP), was meticulously recorded for each participant. Participants underwent obstetric ultrasonography using an Aloka Hitachi Aritta machine (Hitachi Aloka Medical Ltd., Tokyo, Japan) equipped with a 3.5 MHz ultrasound transducer. Fetal biometric parameters, including FKL, biparietal diameter (BPD), femur length (FL), abdominal circumference (AC), and head circumference (HC), were measured using standardized techniques.

Results: GA based on the LMP showed a very strong positive correlation with FKL (r = 0.990, p < 0.001). Strong correlations were also observed with FL, HC, AC, and BPD (all p < 0.001). There was a linear relationship between GA and the FKL.

Conclusion: It can be concluded from the study that FKL is a strong, reliable indicator for determining GA. The high correlation of FKL with GA suggests its potential use in clinical settings for fetal development assessment.

Clinical significance: This study encourages the integration of FKL measurements into routine obstetric ultrasound protocols, alongside traditional parameters like BPD, HC, AC, and FL. There should be the establishment of standardized protocols for measuring kidney length to ensure consistency and accuracy across different practitioners and ultrasound equipment.

## Introduction

Accurately determining gestational age (GA) during pregnancy is paramount for obstetricians and clinicians in managing antenatal care. During the third trimester, when fetal growth is rapid and vital, accurate GA calculation becomes very important [1]. GA was estimated using fetal kidney length (FKL), defined as the average longitudinal length of both fetal kidneys, expressed in millimeters. Research has shown that FKL and GA are strongly positively correlated, especially after the 24th week. For example, one study found a substantial association (r = 0.947) between FKL and GA, with an FKL of 35.66 ± 6.61 mm [2].

Additionally, when paired with traditional fetal biometric data such as abdominal circumference (AC), femur length (FL), biparietal diameter (BPD), and head circumference (HC), FKL improves the overall efficacy of models for estimating GA [2,3]. However, it should be noted that FKL does not entirely replace existing methods but rather enhances them, providing a more precise estimate of GA, especially in situations where traditional methods exhibit declining accuracy [3,4]. Moreover, some studies indicate that FKL remains relatively stable and unaffected by growth disorders, making it a more reliable indicator of GA than other fetal biometric parameters [2-4].

The usefulness of FKL for the estimation of GA in the third trimester was examined in this study. In order to determine the effectiveness and dependability of FKL in this context, it also aims to perform a comparative analysis with other accepted fetal biometric indices. Unlike specific biometric parameters that may plateau or exhibit variability in late gestation, FKL demonstrates consistent and proportional growth throughout the third trimester. This characteristic makes it a promising candidate for accurate GA estimation, especially when considered alongside other biometric indices commonly utilized in prenatal assessment.

Furthermore, an in-depth understanding of the relative effectiveness and accuracy of FKL in GA estimation will be provided by the comparative analysis with accepted fetal biometric indices like FL, HC, AC, and BPD. The goals of this study are to compare the GA obtained from LMP with those derived from FKL and other biometric indices (BPD, FL, AC, and HC); to determine the most accurate parameter, or combination of parameters, for third-trimester GA; and to assess the accuracy of FKL to estimate GA in the third trimester.

## Materials and methods

The current prospective cross-sectional study facilitates the systematic collection of data from pregnant participants in their third trimester. The research was conducted from 2022 to 2024 at the Department of Obstetrics and Gynecology, Acharya Vinoba Bhave Rural Hospital (AVBRH), Datta Meghe Institute of Higher Education and Research (DMIHER), Wardha. Instituitional Ethics Committee of Datta Meghe Institute of Medical Sciences, Wardha issued approval DMIMS(DU)/IEC/2022/110. The sample size comprised well-dated normal pregnant patients attending Obstetrics and Gynecology services at AVBRH Hospital, Sawangi, Meghe, who met the exclusion and inclusion criteria of the study. These criteria ensured the selection of a homogeneous group of pregnant patients without confounding factors that could affect the accuracy of GA estimation using fetal biometric parameters.

Sample size

The sample size was determined using the formula, $n = ((Z&alpha; + Z&beta;)/C)&sup2; + 3$, where n represents the estimated sample size. In this equation, $&alpha;$ denotes the level of significance and $(1&minus;&beta;)$ indicates the study power. The terms $Z&alpha;$ and $Z&beta;$ correspond to the standard normal deviate values for the chosen significance level and power, respectively. The constant C is calculated as $0.5 &times; ln[(1 + r)/(1 &minus; r)]$, where r is the expected correlation coefficient.

The least sample size needed is 97 if the correlation between GA and FKL is at least 0.3 at the 5% level of significance and 85% power. The accuracy of the result increases with an increase in the sample size. We took 100 pregnant women's samples for the study. The actual correlation lies around 0.9. However, the sample size based on it will be less than 10. Hence, the correlation coefficient is assumed to be lower in order to determine the required sample size.

Inclusion criteria

Pregnant women with singleton pregnancies between 28 and 40 weeks of gestation were included in the study. Enrolment was limited to women whose GA and previous menstrual cycle were verified by first-trimester ultrasonography. Uncomplicated pregnancies with no maternal or fetal risk factors that could influence fetal growth were included.

Exclusion criteria

Pregnancies with congenital fetal anomalies, suspected fetal growth restriction, multiple gestations, or uncertain menstrual dates were excluded. Women with medical conditions such as chronic renal disease, pre-eclampsia, diabetes mellitus, chronic hypertension, substance/tobacco use, autoimmune disorders such as lupus and rheumatoid arthritis, gastrointestinal disorders, thyroid disorders, and eclampsia were also excluded. Cases with fetal renal pyelectasis (>4 mm), abnormal renal morphology, or poor visualization of renal margins were not included. Anomalous fetuses were detected through ultrasound.

**Figure 1:**
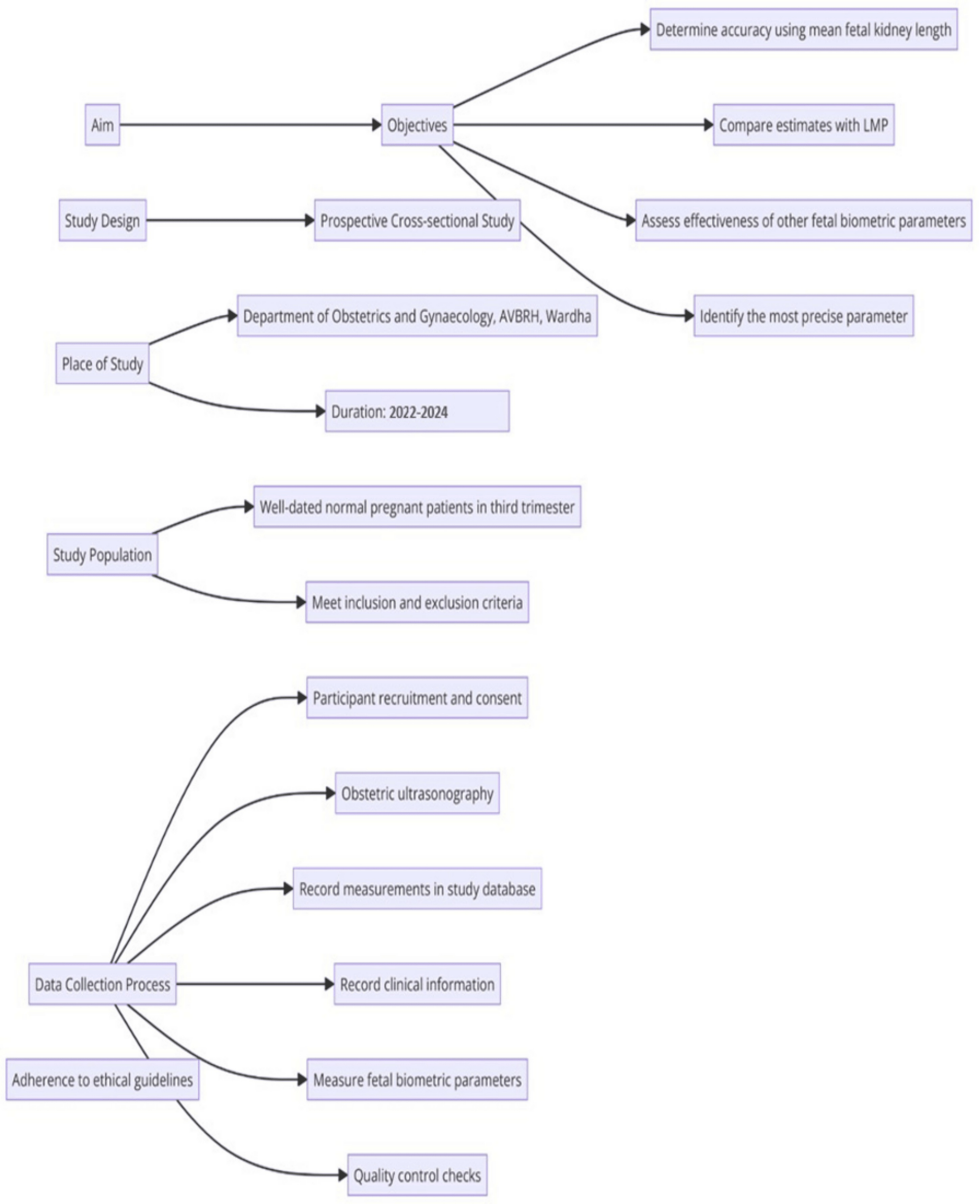
Flow chart of the methodology LMP: last menstrual period; AVBRH: Acharya Vinoba Bhave Rural Hospital

Figure 1 illustrates the methodological workflow of participant recruitment, data collection, and analysis.

Pregnant women meeting the inclusion criteria were approached, provided with detailed information regarding the study, and requested to opt in. Participants who were willing to opt in provided consent papers. For every participant, pertinent clinical data were carefully documented, such as maternal age, parity, medical history, and last menstrual period (LMP). The latest menstrual cycle was used to calculate the reference GA, and first-trimester ultrasonography (less than 12 weeks) was used as the reference standard [5]. Participants underwent obstetric ultrasonography using an Aloka Hitachi Aritta machine (Hitachi Aloka Medical Ltd., Tokyo, Japan) equipped with a 3.5 MHz ultrasound transducer. The examination was carried out in a controlled setting at the AVBRH Department of Obstetrics and Gynecology. Standardized methods were used to measure FKL, BPD, AC, FL, and HC.

Both FKLs were measured in the transverse plane until they were visible immediately below the stomach. The longitudinal axis of each kidney was outlined, and measurements were taken from outer margin to outer margin, excluding the adrenal glands. Careful attention was paid to exclude cases with renal pyelectasis, obscured adrenal and renal border margins, or abnormal renal morphology. To minimize intra-observer variability, each parameter was assessed twice under the supervision of a consultant. Excess gel on the patient's abdomen was removed, and the patient was allowed to adjust her attire for comfort. All measurements were accurately recorded in the study database along with corresponding clinical information. GA derived from FKL was estimated based on the established linear relationship between FKL in millimeters and GA in weeks, approximating a growth rate of 1 mm/week in the last trimester, as supported by previously published literature [6]. Regular quality control checks were performed to ensure the reliability and consistency of measurements throughout the study period. The goal of the data collection procedure was to obtain thorough information for further analysis and interpretation while adhering to ethical standards.

Statistical analysis

All data was entered into Microsoft Excel (Microsoft Corporation, Redmond, Washington, United States) under the guidance of a statistician. Statistical analysis was performed using IBM SPSS Statistics for Windows, Version 25 (Released 2017; IBM Corp., Armonk, New York, United States). Descriptive statistics, including mean and standard deviation, were calculated for each group. The association between variables was assessed using the Pearson correlation test, with statistical significance defined as a p-value < 0.05. By defining GA estimates as accurate if they fell within +/- one week of the reference GA established by LMP, sensitivity and specificity were computed. Each fetal biometric parameter was evaluated independently using this predefined margin of error. Since the main goal was to compare agreement with the reference GA rather than diagnostic discrimination, receiver operating characteristic (ROC) curve analysis was not carried out.

## Results

Eight per cent of the 100 pregnant women were under the age of twenty; 20 to 25-year-olds made up the largest age group, accounting for 46% of the participants. The standard deviation was 4.264 years, while the average age was 25.86 years. Of the 100 expectant mothers, 55% were primigravida and 45% were multigravida (Table 1).

**Table 1:** Baseline characteristics of the study participants

Age (in years)	No. of cases ( n = 100)	Percentage (%)
≤ 20	08	08%
21 – 25	46	46%
26-30	32	32%
31-35	11	11%
36 – 40	02	02%
> 40	01	01%
Area of residency		
Rural	93	93.0%
Urban	07	7.0%
Gravidity		
Multigravida	45	45%
Primigravida	55	55%

Mean ± standard deviation (SD) of FKL and GA estimates using various biometric parameters across LMP-based GA bands in Table 2. FKL grew gradually with increasing GA, exhibiting a near-linear pattern across all GA bands. FKL-derived GA exhibited less variation as compared to other biometric indices, and it closely matched LMP-derived GA, especially in the latter weeks of the third trimester (Table 2).

**Table 2:** Mean ± SD of fetal kidney length and gestational age estimates using various biometric parameters across LMP based gestational age bands SD: standard deviation; FKL: fetal kidney length; AC: abdominal circumference; BPD: biparietal diameter; HC: head circumference; FL: femur length; GA: gestational age; MM: millimeter; LMP: last menstrual period

LMP confirmed gestational age LMP GA(Weeks)	Number of cases	FKL (in mm)	FL GA (weeks)	BPD GA (weeks)	AC GA (weeks)	HC GA (weeks)	FKL GA (weeks)
		Mean ± SD	Mean ± SD	Mean ± SD	Mean ± SD	Mean ± SD	Mean ± SD
28 to 29	5	28.81±0.88	27.98±0.56	30.24±1.58	29.58±1.22	27.80±0.61	28.84±0.88
29 to 30	5	29.73±0.99	29.04±1.10	30.46±1.17	29.34±1.01	28.32±1.67	29.80±1.02
30 to 31	7	30.47±0.11	29.68±1.35	31.29±1.93	29.66±1.53	29.29±2.62	30.51±0.11
31 to 32	6	31.21±0.30	29.91±2.04	30.95±1.53	31.42±1.39	29.82±1.37	31.27±0.21
32 to 33	8	32.13±0.47	31.63±1.36	31.81±1.92	32.20±0.45	30.69±1.56	32.15±0.47
33 to 34	15	33.47±0.69	32.81±1.40	33.35±1.13	33.07±0.86	32.17±1.11	33.54±0.68
34 to 35	11	34.26±0.48	32.85±1.26	34.40±1.28	33.94±0.92	32.97±1.66	34.34±0.41
35 to 36	10	35.39±0.52	34.24±1.44	35.75±1.52	34.82±1.17	33.96±1.80	35.44±0.54
36 to 37	6	36.90±0.81	35.75±1.25	36.22±1.67	36.25±0.48	34.90±1.19	36.92±0.82
37 to 38	7	37.53±0.54	36.24±1.69	36.73±1.50	37.16±1.30	35.96±1.80	37.61±0.54
38 to 39	13	38.98±0.67	37.66±1.33	38.88±1.27	38.54±0.88	37.98±1.34	39.03±0.65
39 to 40	7	39.90±0.81	37.32±1.12	37.87±1.43	38.16±1.49	36.97±0.93	39.97±0.75

**Figure 2:**
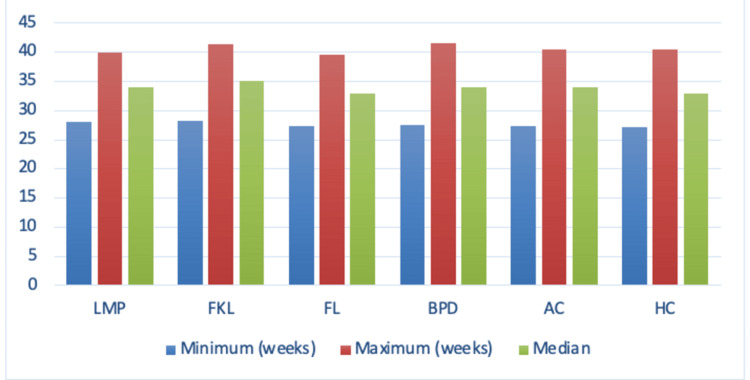
Analysis of variability in gestational age estimates based on different fetal parameters in the study group FKL: fetal kidney length; AC: abdominal circumference; BPD: biparietal diameter; HC: head circumference; FL: femur length; LMP: last menstrual period

With a mean of 34.48 weeks, an SD of 3.21 weeks, and a median of 34 weeks, the GA based on the LMP varied from 28.10 to 40.00 weeks. With a mean of 34.59 weeks, an SD of 3.37 weeks, and a median of 35 weeks, the FKL method derived a GA range of 28.20 to 41.30 weeks. FL-based GA range of 27.30 to 39.50 weeks, with a median of 33 weeks, a mean of 33.40 weeks, and an SD of 3.26 weeks. With a mean of 33.08 weeks, an SD of 3.46 weeks, and a median of 33 weeks, the HC-based GA range is 27.20 to 40.50 weeks (Figure 2).

Table 3 shows the correlation coefficients of FL, HC, AC, BPD, and FKL. LMP GA and FKL GA had the highest correlation (0.990), while LMP GA and BPD GA had the lowest correlation (0.876). The correlation values varied from 0.876 to 0.990. According to this table, all of the relationships are statistically significant because the p-values are less than 0.001. The GA estimations derived from various approaches exhibit a positive linear relationship.

**Table 3:** Correlation coefficient of gestational age based on last menstrual period (LMP GA) with gestational age based on various fetal parameters in the study group FKL: fetal kidney length; AC: abdominal circumference; BPD: biparietal diameter; HC: head circumference; FL: femur length; GA: gestational age; LMP: last menstrual period

Pair	LMP GA Vs BPD GA	LMP GA Vs HC GA	LMP GA Vs AC GA	LMP GA Vs FL GA	LMP GA Vs FKL GA
Karl Pearson Correlation	0.876	0.898	0.942	0.911	0.990
P-value	< 0.001	< 0.001	< 0.001	< 0.001	< 0.001

Table 4 shows the sensitivity and specificity of fetal biometric indices for GA estimation, using LMP-confirmed GA as the reference standard. Sensitivity and specificity were calculated using a predefined accuracy margin of +/- one week. FKL demonstrates the highest sensitivity at 0.91, making it highly effective in correctly estimating GA, and a specificity of 0.80, indicating strong reliability in ruling out inaccurate estimates in the third trimester. FL and AC also exhibit good specificity (0.76 and 0.74, respectively) and high sensitivity (0.87 and 0.85, respectively), ensuring accurate and reliable GA estimations in the third. HC follows with a sensitivity of 0.84 and specificity of 0.72, providing effective identification of the correct GA, although with slightly lower specificity compared to FKL, FL, and AC. BPD shows moderate sensitivity and specificity (0.82 and 0.70), making it less reliable than the other parameters in the third trimester (Table 4).

**Table 4:** Sensitivity and specificity of various fetal parameters in the study group FKL: fetal kidney length; AC: abdominal circumference; BPD: biparietal diameter; HC: head circumference; FL: femur length

Fetal Parameter	Sensitivity	Specificity
FKL	0.91	0.80
BPD	0.82	0.70
FL	0.87	0.76
AC	0.85	0.74
HC	0.84	0.72

## Discussion

The FKL was determined as a potential parameter for estimating GA in the third trimester. Our findings revealed a steady increase in FKL with advancing GA, ranging from 28.15 to 41.25 weeks. Additionally, the FKL across all GAs was calculated as 34.53 ± 3.37 weeks. This suggests that FKL could serve as a reliable indicator of GA, showing a very strong positive correlation (r = 0.990) with GA measured by LMP. Several studies have demonstrated the utility of FKL as a reliable parameter for estimating GA, particularly in the third trimester. Research has consistently shown a strong positive correlation between FKL and GA, which confirms its effectiveness in fetal age assessment. For instance, a study by Khanal et al. demonstrated that FKL has a significant correlation (r = 0.989) with GA, making it a dependable parameter in uncomplicated pregnancies [7]. Similarly, Patibandla and Vasundhara validated the accuracy of FKL in the third trimester, finding it to be more reliable than other common biometric parameters [8]. In another study, Gautam et al. found a very strong positive correlation (r = 0.921) between FKL and GA, indicating that adding FKL to routine fetal parameters improved the accuracy of GA prediction [9]. This study reinforces the potential of FKL to enhance the precision of gestational assessments, especially when combined with other standard measurements. Moreover, Francis et al. highlighted that from 18 to 35 weeks, FKL had a steady growth rate of approximately 1 mm/week, which can be particularly useful for early and late pregnancy assessments [10]. Adding FKL to conventional biometric parameters (BPD, HC, AC, and FL) increased the accuracy of estimating GA and the expected date of delivery. Fathey et al. suggest that FKL can be a valuable tool for determining GA, especially when menstrual dates are not certain, further supporting its reliability and accuracy in clinical practice [11].

In this study, further comparative analysis revealed that FKL had the highest mean GA among all indices, indicating its potential as a precise parameter for GA estimation. Furthermore, the correlation coefficient between FKL and GA measured by LMP was notably higher than that of other biometric parameters, reinforcing the significance of FKL in accurate GA assessment. Farrokh Seilanian Toosi et al. support the assertion that FKL can serve as a precise parameter for GA estimation, indicating its potential over other biometric indices [12]. Khaled Faraag Tawfik et al. conclude that combining FKL with other indices, especially in the late second and early third trimesters, provides a highly accurate estimation of GA [13]. Based on our comprehensive analysis, FKL emerged as the most precise parameter for determining GA in the last trimester. Its strong correlation with GA measured by LMP, consistent increase with advancing GA, and minimal variability make it a promising tool for accurate GA assessment.

A review of recent literature underscores that FKL is a highly reliable parameter for estimating GA, especially in the last trimester. Several studies affirm FKL's robust correlation with GA, demonstrating minimal variability and consistent accuracy. For instance, a study was conducted on FKL as an indicator of GA [14]. Their findings revealed that FKL is a reliable metric, with significant differences between left and right kidney lengths and a strong correlation with GA across various weeks. Similarly, Abo-Donia and Shalaby confirmed these results by developing a regression equation that estimates GA using FKL [15]. This study demonstrated that FKL could predict GA with a high accuracy of ±6.4 days, further establishing FKL as a dependable single parameter for GA assessment.

Limitations

Despite the promising findings, the study had several limitations:

Sample Size and Diversity

The limited sample size may restrict the generalizability of the findings to the broader population. Additionally, most participants in our study were from rural areas, which may limit demographic representation. Therefore, large-scale studies involving diverse populations across different geographic regions are needed to validate and generalize the findings.

Single Center Study

Conducting the study at a single hospital or medical center may have limited the diversity of the participant population and introduced location-specific bias. Multicenter studies involving diverse populations, demographics, and healthcare settings would provide more comprehensive and generalizable data.

Operator Variability 

Inter and intra-operator variability in measuring FKL could introduce measurement inconsistencies, impacting accuracy.

Clinical significance 

The findings of this study indicate that FKL can serve as a valuable adjunct to conventional fetal biometric parameters for estimating GA in the third trimester. Incorporating FKL into routine obstetric ultrasound assessment may enhance accuracy, particularly when traditional parameters show increased variability. Standardised measurement protocols could further improve consistency and reproducibility across different clinical settings.

## Conclusions

The present study demonstrates that FKL is a stable and reliable parameter for estimating GA in the third trimester. Its clinical relevance is reinforced by its predictable growth pattern and strong agreement with GA determined by the LMP. Incorporating FKL alongside conventional biometric measurements may enhance the accuracy of gestational dating, particularly in late pregnancy when standard parameters become less reliable.
